# Olfactory discrimination largely persists in mice with defects in odorant receptor expression and axon guidance

**DOI:** 10.1186/1749-8104-7-17

**Published:** 2012-07-04

**Authors:** Thomas K Knott, Pasil A Madany, Ashley A Faden, Mei Xu, Jörg Strotmann, Timothy R Henion, Gerald A Schwarting

**Affiliations:** 1 Cell Biology Department, University of Massachusetts Medical School, 55 Lake Avenue North, Worcester, MA, 01655, USA; 2 Academic Research Computing, University of Massachusetts Medical School, Worcester, MA, 01655, USA; 3 Institute of Physiology, University of Hohenheim, 70593, Stuttgart, Germany

**Keywords:** Olfactory sensory neurons, β3GnT2, Adenylyl cyclase 3, Axonal convergence

## Abstract

**Background:**

The defining feature of the main olfactory system in mice is that each olfactory sensory neuron expresses only one of more than a thousand different odorant receptor genes. Axons expressing the same odorant receptor converge onto a small number of targets in the olfactory bulb such that each glomerulus is made up of axon terminals expressing just one odorant receptor. It is thought that this precision in axon targeting is required to maintain highly refined odor discrimination. We previously showed that β3GnT2^−/−^ mice have severe developmental and axon guidance defects. The phenotype of these mice is similar to adenylyl cyclase 3 (AC3) knockout mice largely due to the significant down-regulation of AC3 activity in β3GnT2^−/−^ neurons.

**Results:**

Microarray analysis reveals that nearly one quarter of all odorant receptor genes are down regulated in β3GnT2^−/−^ mice compared to controls. Analysis of OR expression by quantitative PCR and *in situ* hybridization demonstrates that the number of neurons expressing some odorant receptors, such as mOR256-17, is increased by nearly 60% whereas for others such as mOR28 the number of neurons is decreased by more than 75% in β3GnT2^−/−^ olfactory epithelia. Analysis of axon trajectories confirms that many axons track to inappropriate targets in β3GnT2^−/−^ mice, and some glomeruli are populated by axons expressing more than one odorant receptor. Results show that mutant mice perform nearly as well as control mice in an odor discrimination task. In addition, *in situ* hybridization studies indicate that the expression of several activity dependent genes is unaffected in β3GnT2^−/−^ olfactory neurons.

**Conclusions:**

Results presented here show that many odorant receptors are under-expressed in β3GnT2^−/−^ mice and further demonstrate that additional axon subsets grow into inappropriate targets or minimally innervate glomeruli in the olfactory bulb. Odor evoked gene expression is unchanged and β3GnT2^−/−^ mice exhibit a relatively small deficit in their ability to discriminate divergent odors. Results suggest that despite the fact that β3GnT2^−/−^ mice have decreased AC3 activity, decreased expression of many ORs, and display many axon growth and guidance errors, odor-evoked activity in cilia of mutant olfactory neurons remains largely intact.

## Background

Understanding the organization of connections in the mammalian olfactory system is a challenge because the rules used to generate a map of axon trajectories from the olfactory epithelium to the olfactory bulb (OB) appear to differ considerably from other sensory modalities, such as the visual and somatosensory systems [1-3]. It is now clear that regulation of adenylyl cyclase activity and generation of cAMP is one of the major contributors of guidance information in olfactory axons [4-6]. In fact, much of the influence that was initially attributed to odorant receptors (ORs) themselves has now been shown to result from OR-dependent cAMP signaling [7]. In this regard, an important question is how olfactory sensory neurons (OSNs) regulate adenylyl cyclase 3 (AC3) activity, since cAMP is responsible not only for activation of the cyclic nucleotide-gated channel (CNG) in cilia but also for downstream signaling in axons via pathways that may regulate the activities of protein kinase A and the transcription factor, CREB [4].

We have previously shown that the glycosyltransferase β3GnT2 is expressed in OSNs and is the key enzyme in the synthesis of polylactosamine (PLN) glycans [6,8,9]. AC3 is heavily N-glycosylated with PLN glycans in OSNs, and its expression is significantly down-regulated on β3GnT2^−/−^ axons [6]. The severe olfactory developmental and axon guidance abnormalities in β3GnT2^−/−^ mice appear in many respects to phenocopy wiring defects described for AC3 knockout mice where olfactory cAMP signaling is also reduced [10-12]. In both models, M72 axons are misguided specifically to multiple heterotypic glomeruli primarily in the ventromedial OB, and P2 axons are mostly lost from the postnatal OB. These changes in axon growth and guidance are due in part to the decrease in cAMP-dependent signaling in both mouse models resulting in altered expression of important axon guidance cues, such as neuropilin-1 and semaphorin-3A [6,13].

Despite these many similarities, the major difference between the two mouse models is their respective abilities to detect and discriminate odors. AC3^−/−^ mice are anosmic due to the absence of cAMP synthesis and the resulting inability to activate CNG channels in dendritic cilia [14]. In contrast, although AC3 expression is significantly reduced in axons extending to the OB of β3GnT2^−/−^ mice, AC3 traffics normally to cilia and although reduced amounts of cAMP are produced, adult β3GnT2^−/−^ mice are not anosmic [6,8]. In order to gain insight into olfactory discrimination in β3GnT2^−/−^ mice, we compared OR gene expression, examined axon trajectories by immunocytochemistry with specific OR antibodies, and carried out an odorant discrimination task on WT and β3GnT2^−/−^ mice. Results presented here show that many OR genes are significantly down-regulated in β3GnT2^−/−^ mice such that only a small number of those axons make connections in the OB. In addition, other OR-specific axon subsets innervate inappropriate targets in the OB. These findings will be discussed in light of the fact that β3GnT2^−/−^ mice can discriminate odors with considerable fidelity.

## Results

### Odorant receptor alterations in β3GnT2^−/−^ mice

Five separate samples of OE were dissected from adult β3GnT2^−/−^ mice and littermate controls and mRNA was isolated from each sample. Following preparation of cDNA and labeling, the samples were each hybridized to an Affymetrix Mouse Gene 1.0 ST Array (Afftymetrix, Santa Clara, CA USA). A median value was calculated for each probe set for each OR gene in the 10 samples. Median values of the five null samples were compared pairwise with median values from the five WT samples. A null/WT ratio was generated that represents the mean of the five pairwise comparisons, Of 1,144 probe sets for OR transcripts, results indicated that mRNAs for 867 of the ORs were expressed at similar levels in WT and β3GnT2^−/−^ OEs, including OR37 and M72. Results also indicated that expression of 256 OR genes was decreased by more than 30% in all five samples obtained from β3GnT2^−/−^ OEs compared to the five WT OE samples. In addition, 21 OR transcripts, including mOR 256–17 and mOR 125–1 were increased by greater than 25% in all β3GnT2^−/−^ OEs. The null/WT ratios of several of the 21 ORs that were increased the most, and a few of the many other ORs that decreased or were unchanged are presented in Table 1. In order to examine these differences in greater detail, we selected several ORs with mean values that were significantly decreased, increased or about the same in β3GnT2^−/−^ OEs compared to littermate controls and performed real time RT-qPCR or *in situ* hybridization analyses.

**Table 1:** **Comparison of odorant receptor gene expression in β3GnT2**^−/−^** and WT OEs**

**Odorant receptor**		**null/WT**
Olfr136	mOR256-7	3.0*
Olfr1370	mOR256-14	2.9
Olfr1259	mOR232-9	2.7
Olfr43	mOR125-1	2.6
Olfr599	mOR23-1	2.5
Olfr1122	mOR264-1	2.4
Olfr131	mOR256-4	2.3
Olfr715	mOR260-1	2.2
Olfr15	mOR256-17	1.9
Olfr156	OR37b	0.9
Olfr160	M72	0.8
Olfr151	M71	0.7
Olfr1264	mOR18	0.6
Olfr2	I7	0.5
Olfr640	mOR13-4	0.4
Olfr17	P2	0.3
Olfr1507	mOR28	0.2

*Median values of the ratios obtained by dividing the gene array signal intensities from β3GnT2 null OEs by the signal intensities from WT OEs.

**Figure 1:**
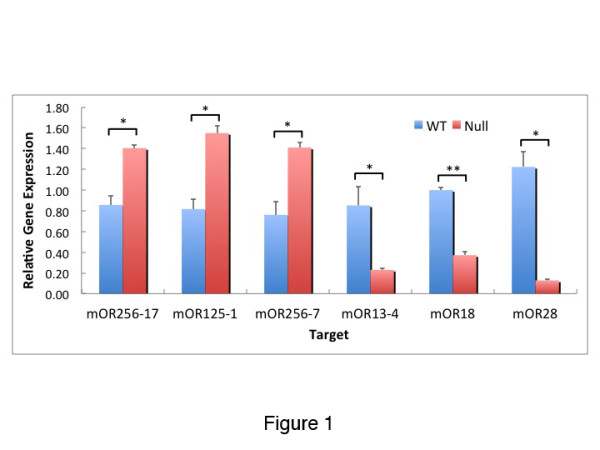
**Quantitative PCR analysis for relative expression of ORs.** In β3GnT2^−/−^ mice the expression of mOR256-17 (Olfr15), mOR125-1 (Olfr43), and mOR256-7 (Olfr136) increased while that of mOR13-4 (Olfr640), mOR16 (Olfr1264), and mOR28 (Olfr 1507) decreased compared to WT mice. Results are the average relative value of three animals normalized to an RNA polymerase 2 reference target. Statistics: Student’s *t* test, **P < 0.001. * < 0.05; mean ± SEM (n = 3).

Using the gene array data as a guide, we chose primer sets for six ORs with null/WT ratios that were either greater than 1.9 or were less than 0.6. After isolating RNA from OE samples, we performed RT-qPCR to find the relative differences in the expression of these ORs (Figure 1). Three ORs, mOR267-17, mOR125-1, and mOR256-7 were analyzed that increased significantly in β3GnT2^−/−^ OEs compared to controls and three ORs, mOR13-4, mOR18, and mOR28 were significantly decreased. qPCR analysis showed that mOR256-17 expression was increased by 71% in β3GnT2^−/−^ OEs compared to controls, and mOR28 was decreased by 89%, validating the gene array generated values of a 90% increase for mOR256-17 and a 79% decrease for mOR28.

**Figure 2:**
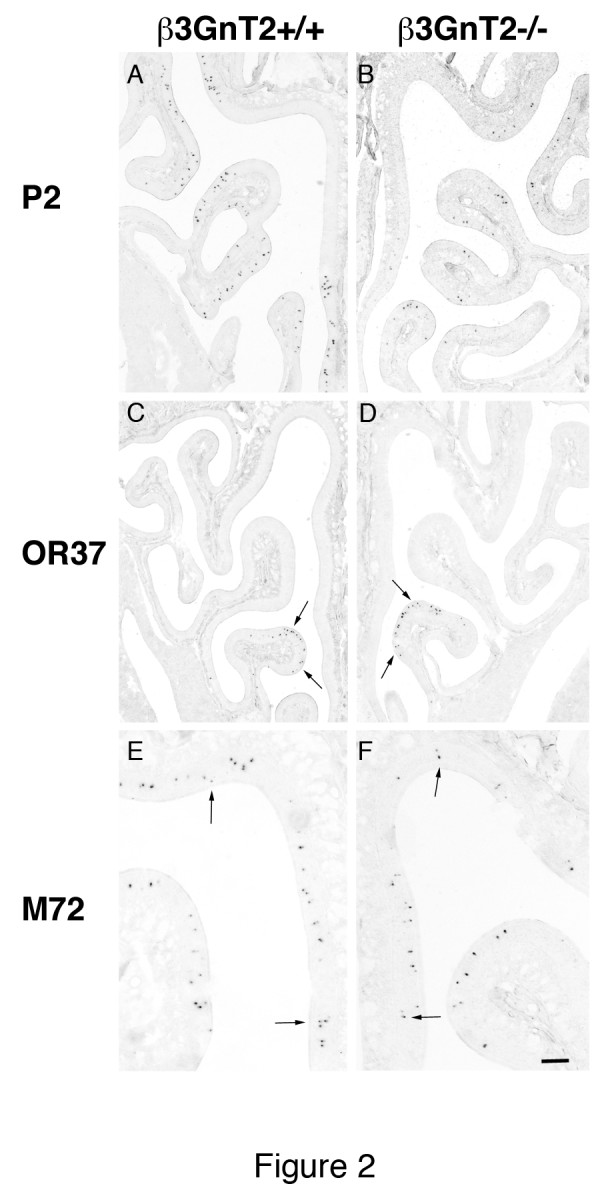
***In situ*hybridization of ORs in the olfactory epithelium of β3GnT2**^**−/−**^**mice.** The expression of some individual odorant receptor mRNAs is decreased in the OE of β3GnT2^−/−^ mice compared to controls. **A, B.** The number of P2 positive cells decreased significantly in β3GnT2^−/−^ mice. **C, D.** The number and position of OR37 expressing cells (arrows) was unchanged in β3GnT2^−/−^ mice. **E, F.** The number and position of M72 expressing cells (arrows) was also unchanged in β3GnT2^−/−^ mice. Scale bar = 200 μM in A-D and 100 μM in E, F.

**Figure 3:**
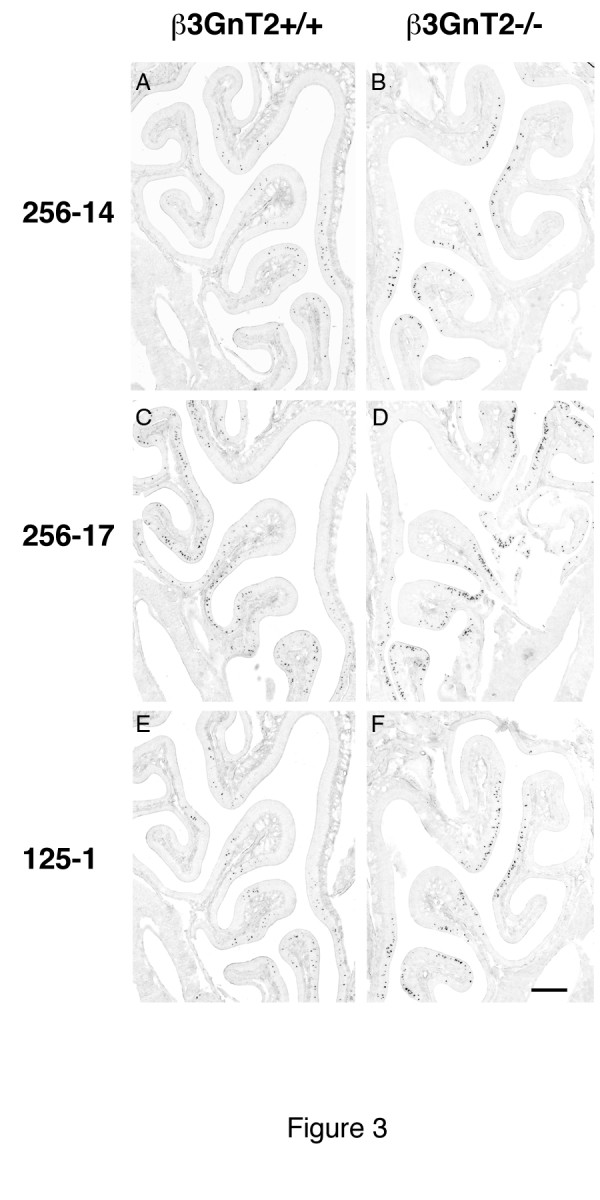
***In situ*hybridization of ORs in the olfactory epithelium of β3GnT2**^−/−^**mice.** Several mRNAs for OR genes are increased in expression in the olfactory epithelium of β3GnT2^−/−^ mice compared to controls. These include mOR256-14, (**A,B**), mOR256-17 (**C,D**) and mOR125-1 (**E,F**). Scale bar in all panels = 250 μM.

As the RT-qPCR results confirmed the gene array analyses that many ORs were either under- or over-expressed in β3GnT2^−/−^ OEs, we made riboprobes to these and other ORs in order to further examine OR gene expression in these mice, and to quantify the changes in the number of OSNs expressing specific receptors. As a control, to confirm a previous observation [8], the number of neurons expressing the P2 receptor was reduced substantially in β3GnT2^−/−^ mice compared to WT. In contrast, the number of cells expressing M72 and OR37 showed no evidence of change (Figure 2, Tables 1 and 2). In no case was the distribution of positive neurons within zones and turbinates changed in β3GnT2^−/−^ mice (arrows in Figure 2). RT-qPCR analysis indicated that several receptors were up-regulated in β3GnT2^−/−^ OEs. *In situ* hybridization studies of these same receptors confirmed that the number of OSNs expressing mOR256-14 (olfr1370), mOR256-17 (olfr15) and mOR125-1 (olfr43) were significantly increased in β3GnT2^−/−^ OEs compared to WT OEs (Figure 3 and Table 2).

**Table 2:** Odorant receptor expression in olfactory sensory neurons

**Receptor**	**β3GnT2+/+**	**β3GnT2−/−**	**% Change**	**p value**
M72 (mOR171-3)	8,162 +/− 1,062	8,008 +/− 1,668	−3.0 +/− 12.1	0.88
olfr15 (mOR256-17)	96,389 +/− 15,250	148,170 +/− 14,500	+59.0 +/− 19.8	0.038
OR37 (mOR262-14)	7,579 +/− 521	7,073 +/− 1,424	−8.5 +/− 12.5	0.65
olfr43 (mOR125-1)	27,896 +/− 3,550	38,339 +/− 3,610	+38.8 +/− 6.2	0.004
olfr1370(mOR256-14)	15,957 +/− 1,833	22,161 +/− 2,183	+39.8 +/− 8.77	0.04
P2 (mOR263-5)	25,377 +/− 1,772	5,396 +/− 535	−78.7 +/− 1.7	0.001

To analyze any differences within a slide series, *In situ* experiments were done using the same probe on two slides from a WT series and two from a null series. These slides were found to have less than 3% differences in receptor counts for either genotype. Thus, the total amount of receptors for each probe is an extrapolation from counts made on approximately 5% of the tissue. Statistics are done with a two-Way ANOVA to account for any variability, which may occur between each *In situ* experiment.

Interestingly, as is the case in rats [15], the number of cells expressing different ORs varies significantly, often by several fold in WT mice. For example, there are more than 10 times more mOR256-17^+^ cells than M72^+^ neurons in the adult OE. However, although our sample is small, changes in expression in β3GnT2^−/−^ mice do not appear to be related to the absolute number of cells expressing a given OR. For example, although their expression levels are about the same in WT mice, the number of mOR125-1^+^ cells increased by about one third in β3GnT2^−/−^ mice, whereas the number of P2^+^ neurons decreased by more than 75% (Table 2).

### Analysis of mOR256-17 axon guidance in β3GnT2^−/−^ mice

**Figure 4:**
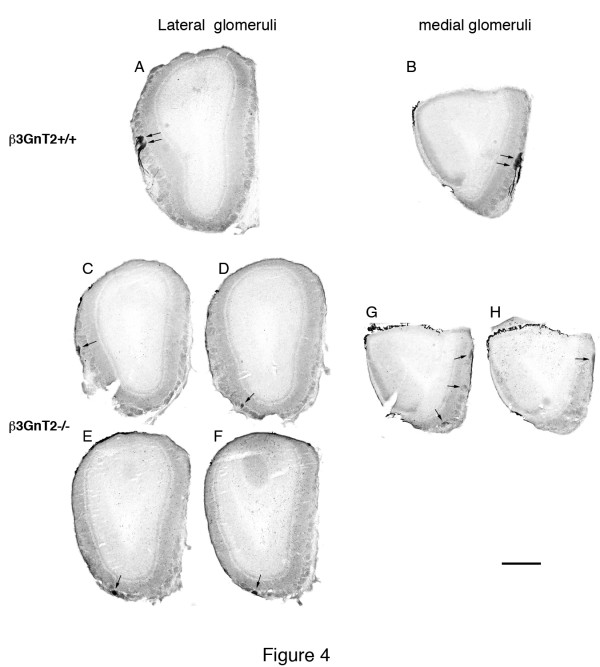
**mOR256-17 Immunocytochemical analysis of the adult olfactory bulb.** In WT mice antibodies to the mOR256-17 OR identify two lateral glomeruli (arrows in **A**) and two medial glomeruli in the extreme caudal OB (arrows in **B**). In β3GnT2^−/−^ mice there are four lateral glomeruli. The most rostral mutant glomerulus (arrow in **C**) is found in its approximate normal position compared to controls. Three additional lateral glomeruli in the null OB are located in an aberrant caudal and ventral position (arrows in **D, E** and **F**) compared to WT glomeruli. In β3GnT2^−/−^ mice, there are three diffuse medial glomeruli, one of which (arrow in **G**) is in the approximate correct position. The other three medial glomeruli in the null OB are in either more ventral or more dorsal positions (arrows in G and **H**) compared to control glomeruli. Scale bar = 500 μM.

Antibodies to the mOR256-17 odorant receptor were used to map the position of glomeruli in adult OBs. In WT mice, the antibody reacted with axons in the nerve layer and glomeruli within a narrowly circumscribed area of the OB. The antibody consistently labeled two glomeruli on the lateral surface of the OB, midway between the dorsal and ventral extent of the OB. The two immunoreactive glomeruli were always positioned side by side (arrows in Figure 4A). Antibodies to mOR256-17 also reacted with axons in the nerve layer and two glomeruli on the medial surface of the OB. These two medial mOR256-17^+^ glomeruli were positioned midway between the dorsal and ventral extent of the OB and were always side by side, identically to the lateral immunoreactive glomeruli (arrows in Figure 4B). In previous studies, immunocytochemical analysis using this same antibody normally revealed just one lateral and one medial glomerulus per OB [16], although other studies have also found two medial and two lateral mOR256-17^+^ glomeruli [17]. It is not clear whether this reveals a second mOR256-17^+^ glomerulus or if the antibody cross-reacts with closely related ORs that normally project to adjacent glomeruli. The total number of mOR256-17^+^ neurons is a extremely high compared to some other ORs (Table 2), thus it would not be surprising if there is normally more than one mOR256-17^+^ glomerulus in each half OB.

In β3GnT2^−/−^ OBs, mOR256-17 immunoreactive glomeruli are present in the OB but in more scattered positions on the lateral and medial surfaces. The mutant OBs are shaped somewhat differently from WT OBs in that they are shorter along the rostrocaudal axis and along the dorsoventral axis but not mediolaterally [8]. In this example, which is typical of four OBs examined, a mOR256-17 immunoreactive glomerulus is present on the lateral surface of the OB (arrow in Figure 4C) in a position similar to those seen in the wild type OB. Three additional mOR256-17 positive glomeruli are also detected in more caudal sections of the ventral OB (arrows in Figure 4D, E, F). Even further caudally, three additional mOR256-17 glomeruli are visible in the ventral OB that appear to represent medially projecting axons (arrows in Figure 4G). One of these mutant glomeruli is in the approximate correct position compared to wildtype OB, but the others are located either more ventrally or more dorsally from their normal position. The mOR256-17 positive glomerulus in Figure 4H is the same very large glomerulus as the more dorsal glomerulus in Figure 4G. The aberrant positions of glomeruli in null mice is very similar from mouse to mouse, just as OR-defined glomeruli are found within a very restricted area of the OB in WT mice. We previously showed that this was also the case for M72 glomeruli [9]. These results suggest that other guidance information is maintained in β3GnT2^−/−^ mice that continues to steer axons to specific but aberrant targets.

**Figure 5:**
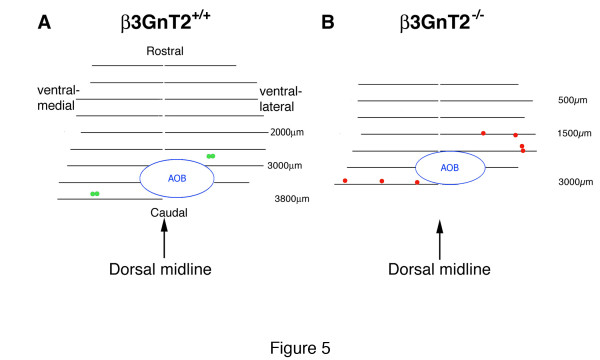
**Composite map of mOR256-17 glomerular positions in adult β3GnT2 mice.** The position of glomeruli in one OB of WT (**A**) and β3GnT2^−/−^(**B**) mice was plotted as a two dimensional map. The OB was flattened and unrolled from the dorsal midline (arrows), and the location of mOR256-17 glomeruli plotted as the relative circumferential distance from the dorsal midline in serial 50 mm coronal sections (horizontal lines) beginning at the anterior extent of the OB. The distance in microns from the anterior tip of the OB is at the right of each plot. Note that the plot of the mutant OB is smaller than that of the WT OB along the anterior-posterior axis.

A map of the positions of mOR256-17 glomeruli in the adult OB clearly shows the difference between the WT and β3GnT2 mutant mouse. The map is a two-dimensional representation of the glomerular layer, looking down at the dorsal surface of the OB after it has been flattened by unrolling it from the ventral midline, rostral is at the top. The map of the mOR256-17^+^ glomeruli in WT OBs (green circles Figure 5A) shows two lateral glomeruli rostral to the AOB, midway between the dorsal and ventral surfaces of the OB and two medial glomeruli close to the caudal end of the OB, midway between the dorsal and ventral surfaces of the main OB.

The map of the β3GnT2^−/−^ OB (Figure 5B) is smaller along the rostrocaudal axis but has similar dimensions to the WT OB along the mediolateral axis. As shown in this map, there are seven glomeruli in total (red circles in Figure 5B), compared with four glomeruli in the WT OB. Although glomeruli are not located in an absolutely fixed position from mouse to mouse, most glomeruli map to within 100 to two hundred microns of the position shown on this map. In addition, three of the mOR256-17 positive glomeruli on the lateral surface of the β3GnT2^−/−^ OBs are near the ventral surface of the OB. On the medial surface of the mutant OB, one of the glomeruli is positioned identically to the WT medial glomeruli, but the other two glomeruli are scattered ventrally and dorsally.

### Axons from under-represented ORs extend into the OB

**Figure 6:**
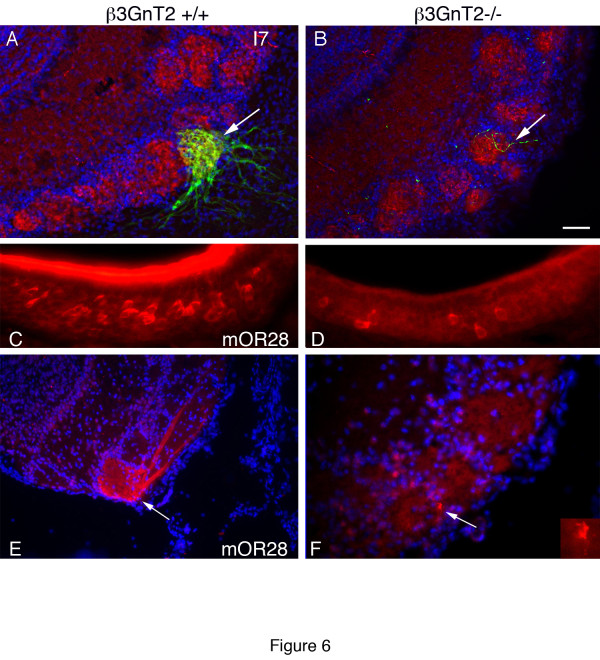
**Reduction in OR expression does not prevent axons from correctly targeting the OB.** I7^+^ axons normally form a medial glomerulus in the OB near the ventral midline (arrow in **a**). In β3GnT2^−/−^ mice, there are insufficient numbers of I7^+^ axons to form a glomerulus but individual axon targets an identically positioned medial glomerulus (arrow). Synaptophysin (red) staining in **A** and **B** suggest that WT and null glomeruli contain functionally active connections. mOR28 is expressed on clusters of neurons in the ventral OE and is heavily expressed on dendrites on the luminal surface of the OE. In null OE’s (**D**), the number of mOR28^+^ neurons is greatly reduced compared to OEs (**C**). mOR28^+^ axons normally form a medial glomerulus in the caudal OB near the ventral midline (arrow in **E**). Very few mOR28^+^ axons can be found targeting this glomerulus in β3GnT2^−/−^ OBs (arrow in **F**, see inset). Scale bar = 50 μM in all panels.

Although the level of expression of many ORs is significantly decreased in the OE of β3GnT2 mice, independent analysis using transgenic mice, *in situ* hybridization or immunocytochemistry reveals that expression of these ORs is not completely lost; rather the number of OR expressing cells may be greatly reduced (Figure 1, Table 2). For example, gene array data showed that the I7 odorant receptor was decreased by more than 50% and *in situ* hybridization and RT-qPCR results indicated that the number of P2 neurons and mOR28^+^ neurons were decreased by 78% and 80% respectively in β3GnT2^−/−^ mice compared to controls. In order to examine the fate of axons expressing under-represented ORs in β3GnT2^−/−^ mice, we studied two OR subsets independently, I7-GFP^+^ axons using GFP fluorescence combined with synaptophysin labeling of presynaptic terminals and mOR28^+^ axons by immunocytochemistry. In WT mice, the medial I7^+^ glomerulus is normally visible near the ventral midline (arrow in Figure 6A). Although we have never seen an I7-GFP^+^ glomerulus in postnatal β3GnT2^−/−^ mice, I7-GFP^+^ axons are detectable in the nerve layer and glomerular layer of adult null mice. In Figure 6B, an I7^+^ axon can be seen entering a synaptophysin^+^ glomerulus (arrow) in approximately the correct location of the β3GnT2^−/−^ OB. However, this glomerulus is for the most part I7 negative but synaptophysin positive, indicating that it is principally populated by axons expressing other ORs.

Gene array analysis showed that mOR28 mRNA was reduced more than any other OR and RT-qPCR results indicated that mOR28 mRNA was significantly decreased in β3GnT2^−/−^ OEs compared to wild type OEs (Figure 1). Immunocytochemical analysis (Figure 6C, D) confirms that the number of mOR28^+^ neurons is decreased by about 75% in β3GnT2^−/−^ OEs. In spite of the severe loss of mOR28 expression in β3GnT2^−/−^ mice, immunoreactive axon projections are visible in the nerve layer of the OB and can be seen targeting glomeruli in the same location that mOR28^+^ axons normally form glomeruli in WT mice (arrows in Figure 6E,F).

### Neuronal activity is unchanged in β3GnT2 null mice

Previous studies have identified two distinct mechanisms for the regulation of OSN gene expression by olfactory signaling reviewed in [7]. One class of genes, including axon guidance cues such as Nrp1 and Sema3A, are directly modulated by cAMP levels established by AC3 [4,13]. For a second class of genes, transcriptional levels are determined by neuronal activity downstream of AC3. This molecular subset exhibits OE expression changes in either naris occluded mice or anosmic cyclic nucleotide gated channel subunit A2 (CNGA2) null mice that are not directly cAMP-dependent [5,17]. For example, microarray analysis studies revealed that RNA levels of the Leucine Rich Repeat Containing 3b (Lrrc3b) gene are decreased more than ten-fold in CNGA2 null OEs relative to WT mice, while those of the calcium binding protein calretinin are elevated nearly four-fold by the loss of activity.

**Figure 7:**
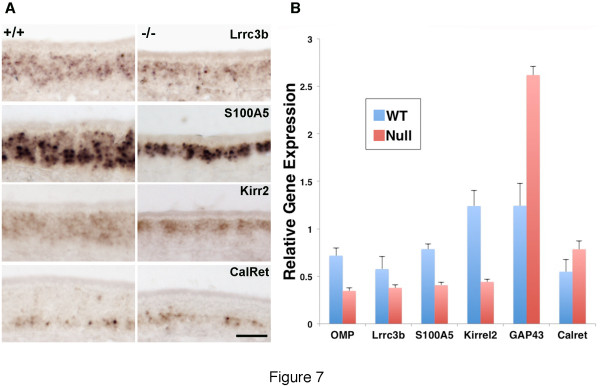
**In situ hybridization of odor-evoked activity dependent genes in the OE.A.** The expression of genes that are regulated by activity-dependent processes were analyzed in adult WT and β3GnT2^−/−^ OEs. Calretinin, which is expressed in immature neurons and is greatly enriched in CNGA2^−/−^ mice is unchanged in expression in β3GnT2^−/−^ OEs. The Leucine Rich Repeat Containing (Lccr3b), S100A5, and kirrel2 genes, which are all expressed in mature neurons in WT OEs and down-regulated in CNGA2^−/−^ mice, are also unchanged in levels of mRNA expression. Note that increased cell death in β3GnT2^−/−^ OEs results in a thinner layer of mature neurons [8]. Scale bar = 50 μM in all panels. **B**. Real-time quantitative PCR analysis of activity-dependent gene expression in microdissected adult wild type and β3GnT2^−/−^ OE samples. Relative levels of Lrrc3b, S100A5 and kirrel2 mRNA in null mice decreases in parallel with the mature neuron marker OMP. Immature neurons expressing GAP-43 correspondingly increase in proportion in the null OE. Consistent with this, calretinin expression in the basal OE is preserved in null mice. Thus, the expression of activity-dependent genes fluctuates only in accordance with the dynamics of the cell population they are expressed in, suggesting that odor-evoked activity is not as severely compromised in β3GnT2^−/−^ mice relative to CNGA2 mutants. Error bars are the mean +/− SEM (n = 3 for GAP-43; n = 6 for all others).

We reasoned that β3GnT2^−/−^ mice should show similar gene alterations if the perturbations in olfactory connectivity and cAMP signaling compromised odor-evoked activity. Surprisingly, this was not the case (Figure 7A), as the activity-dependent genes we analyzed by in situ hybridization were unchanged in β3GnT2^−/−^ mice except for the loss of mature OSNs described previously [6]. Lrrc3b expression is unchanged in OSNs of the thinner null OE, despite the dramatic loss of Lrrc3b evident in CNGA2 null OEs. S100A5, which is down-regulated eight-fold in CNGA2 null mice, remains robustly expressed in β3GnT2 knockouts. Likewise, calretinin expression is unchanged in the basal layer of the β3GnT2^−/−^ OE, despite the fact that its expression is significantly increased and redistributed throughout the OE of CNGA2 KOs [18].

To confirm these results (Figure 7B), we used qPCR to determine expression levels of activity-dependent genes relative to known markers for the neuron populations that undergo dynamic changes in β3GnT2^−/−^ mice. Expression levels of Lrrc3b, S100A5 and Kirrel2 decrease in parallel with those of OMP, a marker for the mature neurons that are lost in β3GnT2^−/−^ mice [6]. Because of the loss of mature OSNs, immature neurons expressing GAP43 increase proportionally in the β3GnT2^−/−^ OE [6]. Despite this, calretinin levels are not significantly altered by the loss of β3GnT2, and there is no redistribution to more apical layers, as reported for CNGA2 knockout mice [18]. These results suggest that neuronal activity in the localized environment of olfactory cilia is not severely compromised in β3GnT2^−/−^ mice compared with CNGA2 null mice.

### β3GnT2 null mice have minor olfactory discrimination deficits

**Figure 8:**
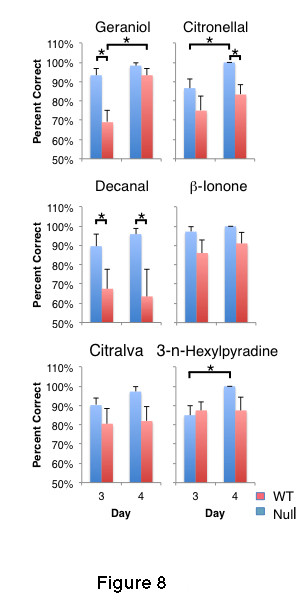
**β3GnT2**^**−/−**^**mice retain the majority of their olfactory discriminatory ability. Mice were tested against pairs of odors for their ability to associate odor with a food reward.** β3GnT2^−/−^ mice can discriminate two of the odors, citralva and β-ionone as well as wild type controls. There is a significant (asterisks) reduction in the ability of null mice to discriminate geraniol, citronellal, decanal, and 3-n-hexylpyridine, but the differences are small and highly variable.

The significant loss of OR gene expression and the axon guidance errors, coupled with the dramatic decrease in AC3 activity reported earlier [6], we expected β3GnT2^−/−^ mice to have a significant loss in olfactory discrimination ability. We, therefore, examined the performance of 9– to 13-week-old β3GnT2^+/+^ and β3GnT2^−/−^ mice in an olfactory discrimination task using structurally unrelated odors. In this assay, mice are trained to associate a food reward with one of two odors. Training took place over a two-day period and testing followed on Days 3 and 4. This test was designed to provide intermittent reinforcement during the first test sessions of Days 3 and 4 so that both wild type and null mice improved their discriminatory ability on Day 4 compared to Day 3 (Figure 8). An initial t-test comparison of the animals with or without a food reward, within a given day, showed no differences on either of the testing days (*P* = 0.24 to 0.47). Therefore, the data within each day were combined and compared using a two-way repeated measures ANOVA using genotype and day as factors (there is no significant statistical interaction between genotype and day, *P* = 0.77).

The analysis shows that β3GnT2^−/−^ mice were able to discriminate all six odor pairings but that they had a significant deficit in their ability to discriminate three of the six odor pairings either on Day 3 or Day 4 compared to littermate controls (asterisks in Figure 8). On Day 4, null mice showed a deficit to two of the six odors, citronellal and decanal, both 10 carbon aldehydes but with several structural differences. Decanal was the only odor to which β3GnT2^−/−^ mice consistently displayed a deficit on both Days 3 and 4. Had the null mice been tested for their ability to discriminate between enantiomers or mixtures of enantiomers, it is likely that further deficits would have been revealed.

## Discussion

Olfactory perception critically depends on the formation of a functional olfactory map in which neurons expressing a given receptor converge on conserved locations in the olfactory bulb. We have previously shown that mice lacking β3GnT2 display abnormal olfactory system development. These mice have significant errors in the guidance of sensory axons to proper targets in the OB [8]. In WT mice AC3 is directly glycosylated by β3GnT2 but in knockout mice this form of polylactosamine (PLN) glycosylation is absent and AC3 enzymatic activity is decreased by 80 to 90% [6]. In order to better understand the role of PLN-glycosylation in olfactory development, we have analyzed the expression of ORs in β3GnT2^−/−^ mice and further examined guidance of specific axon subsets. Results presented here show that in spite of depleted cAMP levels, significant decreases in expression of many ORs, and the presence of many wiring errors, β3GnT2^−/−^ mice have a surprising ability to discriminate odors. These results are in agreement with previous studies suggesting that mutations that alter the organization of the olfactory system may have relatively minor effects on associative behaviors such as olfactory discrimination [19]. The odor-specific patterns of neural activity generated in the OBs of β3GnT2^−/−^ mice are likely to be significantly different from WT controls in many instances, due to changes in OR expression and alterations in glomerular targeting. Although these anatomical perturbations are likely to contribute to the differences observed in our behavioral testing paradigm, the fact that odor discrimination in mutant mice is largely intact suggests that odor-specific patterns of neuronal activity need not be the same in individual animals for them to discriminate odors with significant structural diversity.

### Expression of many ORs is reduced in β3GnT2^−/−^ mice

In this current study, profiling global gene expression in OSNs using microarrays revealed that many other ORs were likely to be misregulated in the β3GnT2^−/−^ OE. Following up on these findings with detailed RT-qPCR analyses, we identified three ORs that were significantly increased in expression in β3GnT2^−/−^ mice and three that were significantly decreased in expression. In order to determine whether these changes were the result of changes in expression levels or of differences in cell number, we performed in situ hybridization studies of six ORs also varying in chromosomal location, and OE zone. The results indicate that mRNA levels for each OR analyzed correlate closely with the number of OR-expressing OSNs that are maintained, suggesting that OR transcriptional levels per neuron are relatively steady. One possible explanation for the changes in expression of these various neuronal populations is that less active neurons may be lost due to competition with more active neurons. Although odor-evoked activity plays a limited role in establishment of the olfactory map, decreased sensory activity can increase the number of heterogeneous glomeruli [20] and activity-dependent competition may contribute to segregation of axons and refinement of connections in the OB [21]. Another possibility is that switching may occur from one OR gene to another[22,23], although we have no evidence that this takes place more frequently in β3GnT2^−/−^ mice than in controls. When an OR gene is deleted, affected OSNs compensate by expressing a different OR, although the replacement receptor would have different binding characteristics [24]. Thus, competition may favor neurons that receive greatest stimulation from a limited repertoire of odorants in their environment.

### Olfactory discrimination is reduced in β3GnT2^−/−^ mice

Although a direct relationship between correct axon targeting and olfactory discrimination has not been established, several studies have shed considerable light on this issue. On the one hand, rats retained most of their olfactory discrimination following surgical removal of significant portions of the target [25]. In contrast, accurate axon targeting in the DII region of the OB appeared to be required for responsiveness to aversive cues [26]. Several other strains of transgenic mice with reduced OR expression or neuronal activity have been tested for their ability to discriminate odors. Using a water reward method to distinguish paired odors, M71 transgenic mice, which under-express many ORs, were able to discriminate between structurally unrelated odors and even between enantiomers but were unable to distinguish mixtures of enantiomers [19]. However, it is not clear how much discriminatory ability is conferred by the over-represented OR, M71, as opposed to the under-expressed ORs [19]. Similarly, although AC3 knockout mice are completely anosmic, AC3 heterozygous mice were shown to have a significant olfactory discrimination deficit [14]. Using either an olfactory avoidance method or a food reward-based test, they showed that AC3 heterozygous mice could discriminate lilial and citralva approximately 50% as well as WT mice, commensurate with a 50% reduction in the levels of AC3. β3GnT2^−/−^ mice express only 10 to 20% of the WT levels of AC3, have reduced levels of OR expression and also axon guidance defects as shown here and previously documented [6,8]. We, therefore, tested β3GnT2^−/−^ mice for their ability to discriminate odors using the same food-based reward paradigm that revealed a deficit in AC3 heterozygous mice. Due to the severe nature of the anatomical, biochemical and signaling deficits seen in β3GnT2^−/−^ mice, we tested odors that were structurally dissimilar (although geraniol, citralva and citronellal have some common features) but had similar adenylyl cyclase stimulatory capabilities. Furthermore, some of the odors tested were ones that had previously been tested in AC3 transgenic mice. For example, AC3 heterozygous mice display a gene-dose dependent decrease in ability to discriminate citralva [14], suggesting that olfactory discriminatory ability is directly related to the levels of AC3 protein in cilia. In contrast, discrimination of citralva by β3GnT2^−/−^ mice was not statistically different from WT mice. Although AC3 is decreased on olfactory axons in β3GnT2^−/−^ mice, the levels of AC3 protein in residual OSN cell bodies and cilia is relatively normal [6], suggesting that signaling required for neuronal activity is largely maintained in null OSN cilia.

### Additional examples of axon guidance errors in β3GnT2^−/−^ mice

We show here, in addition to the changes in OR expression, further examples of aberrant axon guidance in β3GnT2^−/−^ mice. Using immunocytochemical techniques, we showed that mOR256-17^+^ axons are mistargeted to multiple glomeruli on the medial and lateral surfaces of the OB. However, some mOR256-17^+^ axons form a small glomerulus very close to where their normal target should be located. Furthermore, even though only a few I7^+^ and mOR28^+^ axons innervate glomeruli in the adult OB, these axons never reach the level required to form their own unique pair of glomeruli. It is possible that just a small number of OR-specific axons connecting to mitral or tufted cells in the correct targeting region of the OB is sufficient to transduce a signal that is correctly interpreted in the olfactory cortex. These results suggest that although a smaller number of neurons expressing a given OR will likely decrease the probability that an odorant-binding event will lead to perception of that odor, only a small number of connections may actually be required for successful transduction [27].

### Odor-evoked activity is maintained in β3GnT2^−/−^ mice

Olfactory axon guidance is orchestrated via a signaling cascade that includes G protein-coupled receptors, AC3 activation, cAMP synthesis and a downstream pathway involving PKA and CREB [4,28-30]. Thus, activation of AC3 is the key regulator of signaling pathways that subsequently control expression of a number of axon guidance and adhesion molecules, including ephrins, kirrels, neuropilin-1, Sema3A and BIG2 [6,29,31,32]. Importantly though, we show here that odor-evoked activity dependent gene transcription is unaffected in β3GnT2^−/−^ OEs, further supporting the possibility that AC3 activity is preferentially preserved in cilia of these null mice. Interestingly, in many CNS neurons and glia where β3GnT2 is not expressed, AC3 expression is restricted to primary cilia [33]. AC3 is one of several proteins that are actively transported into cilia by a family of proteins associated with renal cystic disease, MKS1 and 3 [34]. Tectonic1, a member of a family of signal-sequence-containing proteins that forms a membrane complex with MKS and other proteins, is required for the ciliary localization of AC3 [35]. OSNs appear to express AC3 differently than CNS neurons and most other cells. Although AC3 is heavily concentrated in WT olfactory cilia, it is also expressed in cell soma and on axons [6,12]. In the absence of PLN-glycosylation, AC3 expression on axons is greatly reduced, although the normal levels of AC3 detected in OSN cells bodies and cilia in β3GnT2 ^−/−^ OEs suggest that the remaining AC3 may preferentially localize to cilia [6]. This would support a hypothesis that a role for β3GnT2 is to ensure that a significant percentage of AC3 protein is not transported to cilia in OSNs, rather than PLN on AC3 and other surface glycoproteins is responsible for maintaining their axonal expression. In fact, all of the components of this canonical transduction process are expressed in axons and it has been suggested that local cAMP synthesis could control expression of guidance proteins specifically in axons [36].

## Conclusions

Olfactory discrimination testing shows that β3GnT2^−/−^ mice have a modest deficit in their ability to distinguish odors. This is in spite of the fact that hundreds of ORs are underrepresented in the OE and many probably do not innervate normal glomeruli in the OB. Furthermore, axon guidance in β3GnT2^−/−^ mice is aberrant, as shown by the abnormal position and increased number of mOR256-17^+^ glomeruli in the OB of knockout mice. Moreover, we have previously shown that AC3 activity is decreased by 80 to 90% in β3GnT2^−/−^ mice, such that guidance and adhesion molecules such as neuropilin-1 and kirrel2 are poorly expressed in axons [6].

The β3GnT2^−/−^ mouse is a unique model of reduced cAMP production in which most of the residual AC3 activity appears to be concentrated in cilia where odor-evoked transduction is largely intact. In contrast, cAMP-dependent transcriptional regulation required for proper axon guidance is virtually absent. An important question that remains unanswered is to what degree glomerular heterogeneity can be tolerated before olfactory discrimination is significantly compromised. Results presented here would suggest that the mouse olfactory system may have a large capacity to withstand down regulation of cAMP signaling, significant deletions in the OR repertoire, alterations in guidance molecule expression and errors in wiring before suffering significant losses of olfactory perception.

## Methods

### Animals

Wild type and β3GnT2^−/−^ mouse littermates were generated from β3GnT2 het/het crosses. PCR was used to genotype the offspring, with a 700-bp fragment amplified from the wildtype β3GnT2 allele and a 1,071–bp fragment amplified from the β3GnT2^−/−^ allele. β3GnT2^−/−^ mice were established from the KST308 embryonic stem cell line, which harbors a secretory trap vector insertion in the β3GnT2 coding sequence as previously described [8,37]. Mice were housed according to standard National Institutes of Health and institutional care guidelines, and procedures were approved by the University of Massachusetts Medical School Institutional Animal Care and Use Committee (Worcester, MA, USA).

### *In situ* hybridization

Digoxigenin (DIG)-labeled sense and antisense riboprobes were transcribed from cDNAs containing subcloned OR coding sequences using vector-specific RNA polymerases and DIG labeling mix (Roche Molecular Biochemicals, Pleasanton, CA, USA). For in situ analysis of adult mouse OEs, age P28 mice were deeply anesthetized and transcardially perfused with 4% Formaldehyde in 0.1 M phosphate buffer (pH 7.4). Brains were removed and further fixed overnight in 4% Formaldehyde at 4°C followed by immersion in 30% sucrose. Bone thickness in the posterior region of the snout from the orbital area to the OB was reduced using surgical knives. The dissected samples were frozen on dry ice, coronally sectioned at 20-μM thickness with a cryostat, and then mounted on Superfrost plus microscope slides, (Thermo Fisher Scientific, Waltham, MA USA). After prehybridization and hybridization, the DIG-labeled RNA hybrids were detected with an anti-DIG Fab fragment conjugated to alkaline phosphatase (Roche Molecular Biochemicals) at a dilution of 1:1,000 overnight at 4°C. The color reaction was produced with nitro blue tetrazolium chloride and 5-bromo-4-chloro-3-indolyl phosphate with levamisole added to block endogenous phosphatase activity.

### Histology and Immunocytochemistry

Tissue was prepared by transcardial perfusion using 2% PLP (paraformaldehyde-lysine-periodate) fixation in 0.1 M phosphate buffer (PB), pH 7.4. Antibodies to mOR256-17 are sensitive to fixation conditions and we determined empirically that immunoreactivity on frozen tissue sections was enhanced using 2% PLP compared to 4% paraformaldehyde or other stronger fixatives. Heads were subsequently removed and postfixed 16 hrs in the same fixative solution, followed by cryoprotection in 30% sucrose. After embedding, tissue sections were prepared on a Microm HM505E cryostat at 50 μM thickness and were then immediately thawed in transwell boats filled with PBS for staining as free floating sections. Tissues were blocked for one hour in 2% BSA, then incubated overnight at 4°C with the primary antibody for mOR156-17 [16] or mOR28 [38] and diluted in 1% BSA/PBS/0.3% Triton X-100. After washing, tissue sections were further incubated for two hours at room temperature with species-specific secondary biotinylated antibody and visualized with the Vectastain ABC peroxidase kit and 3,3’-diaminobenzadine tetrahydrochloride (Vector Laboratories, Burlingame, CA USA), or with Cy3-conjugated (Jackson Immunoresearch, West Grove, PA USA) or Alexa Fluor 488 (Invitrogen, Grand Island, NY USA) conjugated secondary antibodies. Images were captured using a Zeiss Axioplan photomicroscope equipped with a Spot RT camera (Diagnostic Instruments (Sterling Heights MI USA).

### Cell counts

Specific OR-expressing neurons in PD28 OEs of β3GnT2^−/−^ and WT control mice were quantified using a Nikon Eclipse E400 microscope (MVI, Inc., Avon MA USA) at 100X. The complete OE cavity was serial sectioned at 20 μM and collected on 20 to 22 slides per case. Each *in situ* hybridization experiment included a pair of β3GnT2^−/−^ and WT slides for each OR probe. Each probe was used on three different pairs of mice. All the sections from one slide of each genotype were counted and used for analysis. These counts were compared using a paired two-way ANOVA to control for any variability, which may occur between each *in situ* experiment. In addition, pairs of slides within a case, using one probe, were counted and compared and found to be less than 3% different, which was not significant (NS). Thus, we extrapolated the total amount of labeled cells for each OR by adjusting the counts to the total number of slides in each case (Table 2). Statistical analysis was performed using SigmaStat 2.0 (Systat software, San Jose, CA USA**).**

### Real time reverse transcriptase quantitative PCR

RNA was extracted from microdissected olfactory epithelia of 6 β3GnT2^+/+^ and β3GnT2^−/−^ mice at six months of age using Trizol Reagent (Invitrogen), and cDNA prepared as described previously (Henion *et al*., 2011). Quantitative PCR (qPCR) was performed with oligonucleotides designed using online Primer3 software (version 0.4.0). Primer sequences used for template amplification are available upon request. Samples were amplified in triplicate using GoTaq Polymerase (Promega, Fitchburg, WI, USA) in a StepOnePlus Real-Time PCR System (Applied Biosystems, Life Technologies, Grand Island, NY, USA). Relative gene expression levels were determined by the Comparative C_T_ (threshold cycle) method after normalization to RNA polymerase 2 as an endogenous reference.

### Gene array hybridization

OEs from adult mice were collected in PBS on ice. Tissue samples were homogenized using TRIzol (Invitrogen, Carlsbad, CA, USA) and the RNA was collected in the aqueous phase after chloroform extraction. Subsequently, Isopropyl Alcohol was used to precipitate the RNA, followed by washing with 75% ethanol, air-dried, and redissolved in RNAse-free DEPC H2O. After this RNA isolation, a Qiagen RNeasy mini kit (Qiagen, Valencia, CA, USA) was used to further purify the RNA, and the yield analyzed with a DU 650 Spectrophotometer (Beckman Coulter Inc, Brea, CA, USA). Yields per mouse OE measured at an absorbance of 260 nm were 10 to 20 ug RNA.

Affymetrix gene array technology was used to compare the gene profiles between β3GnT2 WT and mutant mouse strains. Individual samples were hybridized to 12 gene chips in a high-density oligonucleotide array (Affymetrix Mouse Gene 1.0 ST Array, 28,853 mouse genes (1,100 OR genes)/chip) that uses 25-mer probes distributed across the transcribed regions of each gene, with a median of 26 probes per gene. The Ambion WT Expression kit (Applied Biosystems, Foster City, CA, USA) was used to synthesize first and second strand cDNA, and generate the purified sense strand for the Affymetrix gene chip WT Terminal labeling kit (Affymetrix, Santa Clara, CA, USA). The single stranded cDNA produced by the Ambion kit was fragmented and labeled using the GeneChip WT Terminal Labeling Kit. The product was then hybridized to the chip for 16 to 17 hours at 45°C, washed, stained and scanned using the Affymetrix GeneChip Array Scanner. The log based signal values were generated using the RMA algorithm in Expression Console. For each pair of samples, results of each probe were compared and an “R” value was calculated. The median of the “R” values of each probe set (R_median) was used for filtering the potentially differential gene expression. No normalization was conducted.

### Olfactory discrimination task

β3GnT2^+/+^ and β3GnT2^−/−^ mice 9 to 13 weeks old were trained in an olfactory discrimination task [39]. Pairs of odors, each known to elicit strong cAMP responses were used in the sand buried food task to measure an animal’s ability to associate an odor with a reward [40]. The mice were food-deprived for 24 hours before the first day of testing began then maintained on a restricted diet throughout the four day trial. On Day 1, the mice were allowed to locate a food reward in the sand. Day 2 was a training day in which each mouse was presented with a food reward in the sand, which was associated with a test odor. The test odors were shifted to different positions within the cage during the training and subsequent testing days. The training period consisted of six 20-minute time periods with 10-minute intervals, where each mouse would have an opportunity to locate the food reward. Early in the training the mice were allowed additional time, if necessary, to locate the food reward. On Day 3, mice were given a choice between a novel odor and the same test odor presented on Day 2. Subsequent sets of six trials each on Days 3 and 4 were scored either correct or incorrect accordingly to which dish each mouse would begin digging. Days 3 and 4 consisted of two separate sets of trials. In the first set of trials the mice were tested without a buried food reward, while in the second set of trials the reward was present.

## Abbreviations

AC3: Adenylyl cyclase 3; CNG: Cyclic nucleotide gated channel; CREB: Cyclic nucleotide response element binding protein; DIG: Digoxigenin; OB: Olfactory bulb; OE: Olfactory epithelium; OR: Odorant receptor OSN, olfactory sensory neuron; Nrp1: Neuropilin-1; PBS: Phosphate-buffered saline; PKA: Protein kinase A; PLN: Polylactosamine; RT-qPCR: Reverse transcriptase quantitative polymerase chain reaction; Sema3A: Semaphoring 3A; WT: Wild type.

## Competing interests

The authors declare that they have no competing interests.

## Authors’ contributions

TKK designed most of the experiments and drafted the initial manuscript. PAM and AAF performed the gene array and RT-qPCR experiments. MX analyzed the gene array data. JS provided the 256–17 antibody and helped to revise the manuscript. TRH supervised the molecular analyses and helped to write and revise the manuscript. GAS supervised the whole study and edited the manuscript. All authors read and approved the final manuscript.
